# Reversal of cerebrovascular constriction in experimental cerebral malaria by L-arginine

**DOI:** 10.1038/s41598-018-34249-2

**Published:** 2018-10-29

**Authors:** Peng Kai Ong, Aline S. Moreira, Cláudio T. Daniel-Ribeiro, John A. Frangos, Leonardo J. M. Carvalho

**Affiliations:** 1 La Jolla Bioengineering Institute, La Jolla, CA USA; 2 Laboratory of Malaria Research, Instituto Oswaldo Cruz, Fiocruz, Rio de Janeiro, Brazil

## Abstract

Vascular dysfunction associated with low nitric oxide (NO) biavailability and low plasma L-arginine levels is observed in both human and experimental cerebral malaria (ECM). In ECM, cerebrovascular constriction results in decreased pial blood flow and hypoxia, and administration of NO donors reverses constriction and increases survival. Supplementation of L-arginine, the substrate for NO synthesis by NO synthases, has been considered as a strategy to improve vascular health and act as adjunctive therapy in human severe malaria. We investigated the effect of L-arginine supplementation on pial vascular tonus of mice with ECM after direct superfusion on the brain surface or systemic delivery. Pial arteriolar diameters of *Plasmodium berghei*-infected mice with implanted cranial windows were measured using intravital microscopy methods, before and after L-arginine administration. Systemic delivery of L-arginine was performed intravenously, at 10, 50, 100 and 200 mg/kg, as bolus injection or slowly through osmotic pumps, combined or not with artesunate. Direct superfusion of L-arginine (10^−7^M, 10^−5^M and 10^−3^M) on the brain surface of mice with ECM resulted in immediate, consistent and dose-dependent dilation of pial arterioles. ECM mice showed marked cerebrovascular constriction that progressively worsened over a 24 h-period after subcutaneous saline bolus administration. L-arginine administration prevented the worsening in pial constriction at all the doses tested, and at 50 mg/kg and 100 mg/kg it induced temporary reversal of vasoconstriction. Slow, continuous delivery of L-arginine by osmotic pumps, or combined bolus administration of artesunate with L-arginine, also prevented worsening of pial constriction and resulted in improved survival of mice with ECM. L-arginine ameliorates pial vasoconstriction in mice with ECM.

## Introduction

L-arginine is an essential amino acid substrate required for the enzymatic production of nitric oxide (NO) by NO synthase (NOS) and its deficiency can impair the NO synthesizing machinery^1^. NO, being a physiologically important signaling molecule ubiquitously expressed in biological systems, can exert profound effects on the regulation of vascular function including blood shear stress-mediated smooth muscle relaxation and vasomotor tone, endothelial adhesion molecule expression, as well as platelet activation and aggregation^2^. Hypoargininemia, a condition described by L-arginine deficiency, is a common pathological hallmark shared by both human cerebral malaria (CM) and murine/experimental CM (ECM)^3–6^, and has been associated with a high rate of CM case-fatality^3^. The mechanisms behind hypoargininemia are poorly understood, but recent studies have shown that plasma L-arginine depletion is driven primarily by a decreased rate of L-arginine, citrulline and ornithine appearance, and decreased conversion of citrulline to L-arginine^5^. Both hypoargininemia and low NO bioavailability have been intimately linked to the pathogenesis of CM^3,7^ and ECM^4^, which is characterized by a state of cerebrovascular dysfunction depicted by vasoconstriction, impaired vasodilation, inflammatory cells (e.g. leukocytes and platelets) adherence, endothelial damage, vascular leakage, haemorrhages, acidosis and hypoxia^8–10^. L-arginine supplementation aimed at restoring NOS production of NO appeared to be capable of ameliorating endothelial dysfunction in severe malaria^11,12^, and could thus be potentially applied as adjunctive therapy for CM/ECM treatment^13^. It may also be possible that L-arginine supplementation can be beneficial to cerebrovascular health by enhancing blood rheological properties in microvessels, for instance, through improving flow deformability of red cells infected by the malarial parasites^14^.

The correlation between an adjunctive therapy involving L-arginine aimed at restoring vascular health and its efficacy of treating CM in patients appeared unclear. Following initial promising results^11,12^, a clinical trial failed to demonstrate a benefit of L-arginine in severe malaria despite safety of the treatment^15^. That study however did suggest an inadequacy of the L-arginine dosage administered, and recommended increasing the L-arginine dosage to achieve the desirable pharmacological effects of improving lactate clearance, restoring endothelial functionality and enhancing treatment efficacy. Pharmacokinetics studies of L-arginine administration in patients with moderately severe malaria have been performed^16^ and a pharmacokinetic-pharmacodynamic model has been developed^17^, and indicated that recovery of endothelial function might increase with increased infusion duration, and the percent time achieving therapeutic response increases with increasing L-arginine dose. In these studies, reactive hyperemia-peripheral arterial tonometry (RH-PAT) index was used as a validated measure of endothelial function and organ perfusion, and showed high variability in the malaria patients. In the case of CM patients, acquiring an understanding of the L-arginine pharmacodynamics in terms of its cerebrovascular effects in response to different dosing regimens would be necessary to ensure optimal delivery of this pharmacological agent in eliciting the desired outcome with maximal potency. In the mouse model, preventative L-arginine administration did not significantly decrease the incidence of ECM^5,18^ and high doses actually resulted in decreased survival^19^. It should be cautioned that these studies did not verify whether those administered schemes and dosages exerted an effect on resuscitating the impaired cerebrovascular response in ECM. In other words, without an assessment of cerebrovascular function in the murine brain, it remained elusive whether various modes of systemic L-arginine administration (bolus/continuous delivery) or various dosage strengths tested are capable of delivering pharmacologically effective amount of this agent to the brain to restore impaired cerebrovascular reactivity and possibly avert the fate of ECM development. The above should be further emphasized since exogeneous sources of L-arginine supplementation would be inevitably susceptible to systemic degradation in the presence of intense *in vivo* inflammation associated with the malarial infection^20^. In addition, and importantly, the above-described treatment regimes refer to ECM-preventative strategies; the effect of L-arginine administration as adjunctive therapy in mice with established ECM in combination with antimalarial drugs has not been shown.

A cranial window superfusion method enabled local delivery of test compounds to the highly delicate brain tissue of living mice with ECM^21^, leading to the discovery that mice with ECM show impaired endothelial and neuronal NOS (eNOS and nNOS)-mediated cerebrovascular dilatory responses, which were partially restored by tetrahydrobiopterin (BH4) superfusion^22^. In the present study, this approach was used to deliver L-arginine to the brain in order to assess the cerebrovascular reactivity to L-arginine so that systemic intravascular sources of L-arginine degradation under an ECM setting can be minimized. In addition, by adopting a classical closed cranial window model for visualization and monitoring of the cerebrovasculature during a treatment regime, we seek to develop an understanding on how cerebrovascular reactivity in ECM mice could be influenced by different systemic routes and regimes of L-arginine administration. In particular, we monitored reactivity of arterioles in terms of their diameter change since these small resistive vessels can react dynamically to the application of vasoactive agents which is critical to cerebral blood flow regulation. When an L-arginine delivery scheme that can produce a significant beneficial effect on cerebrovascular tone (e.g. based on reversing vasoconstriction by the greatest extent) in ECM was available, we further investigated whether this effect was sustainable when applied in an adjunctive therapy setting with the anti-malarial drug, artesunate. Our findings demonstrated a dose-dependent effect of L-arginine exposure on the recovery of impaired cerebrovascular reactivity upon direct delivery to the brain tissue, however with variable responses following systemic administration, in combination or not with artesunate treatment.

## Results

### Parasitemia, motor score and temperature

ECM mice that were used in the experiments were hypothermic, with rectal temperatures ranging between 32 °C and 36 °C (uninfected controls: 39.1 ± 0.5 °C). They also presented decreased motor scores (10 ± 1.1; uninfected controls: 22.0 ± 0.6). At the time of ECM, mice showed mean parasitemia of 13.9 ± 3.6%).

### Pial arteriolar diameter responses to L-arginine superfusion

**Figure 1:**
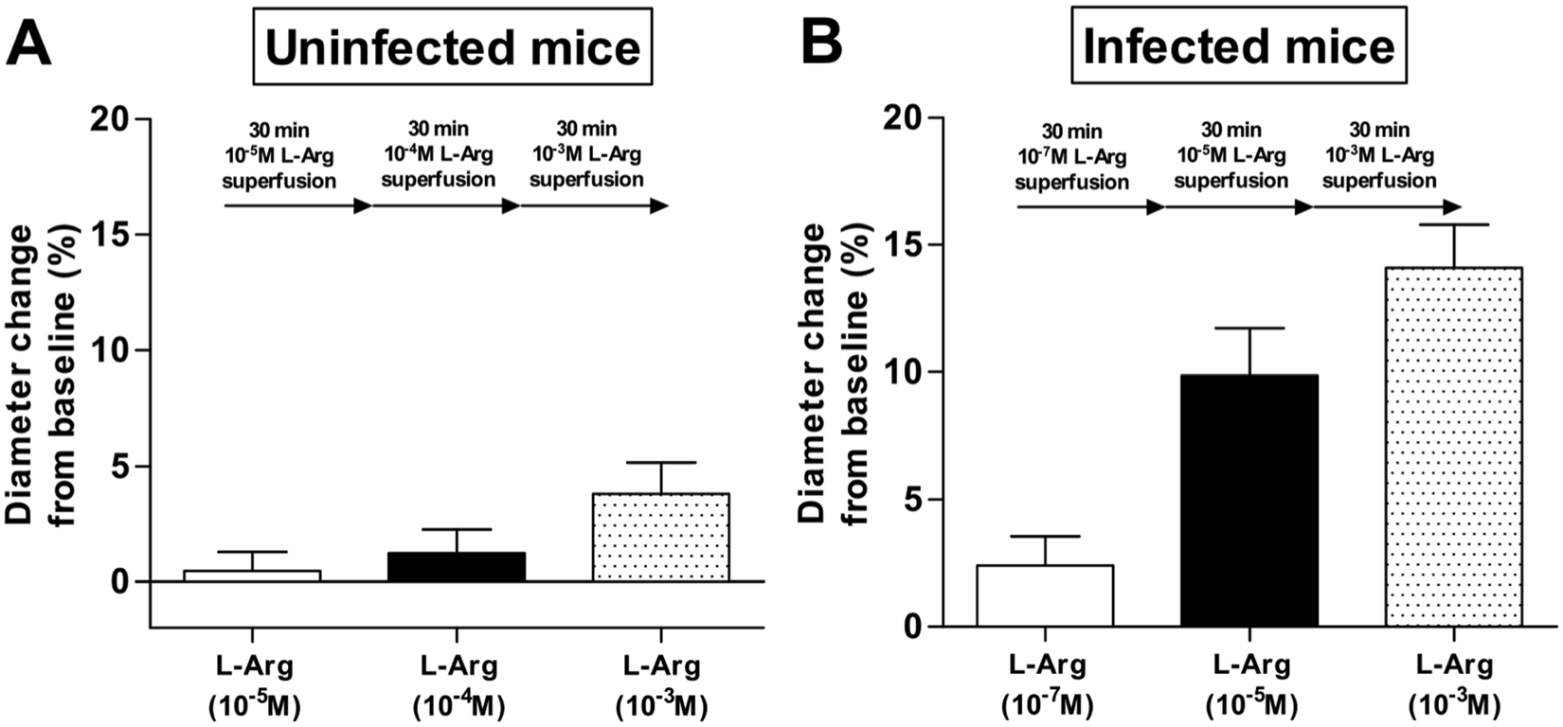
Pial arteriolar diameter responses to L-arginine superfusion in uninfected (**A**) and infected (**B**) mice. L-arginine was introduced in incremental concentration (10^−5^, 10^−4^ and 10^−3^M for uninfected mice and 10^−7^, 10^−5^, 10^−3^M for ECM mice), with a superfusion duration of 30 min for each concentration. Four mice were investigated in each group. Total number of arterioles examined are 24 and 17 in (**A**,**B)**, respectively. Diameter changes are presented as a percentage of pre-treatment baseline. L-arginine superfusion at 10^−5^ and 10^−3^M induced significant (p < 0.05) increases in arteriolar diameters in ECM mice (**B**).

Control experiments were performed in uninfected mice to examine their pial arteriolar reactivity to the superfusion of L-arginine. As shown in Fig. 1A, arteriolar diameters were relatively unchanged from their baseline over a range of L-arginine concentrations tested (10^−5^M to 10^−3^M), implying that L-arginine supplementation (direct delivery at the brain surface) has minimal effect on arteriolar vasomotor tone in healthy mice.

The pial arterioles of mice with ECM (day 6 of infection) were suffused with increasing concentration of L-arginine (from 10^−7^M to 10^−5^M to 10^−3^M), with a superfusion duration of 30 minutes for each concentration to ensure steady state arteriolar diameters were obtained before the next higher concentration of L-arginine was delivered. A dose-dependent increase in arteriolar diameter from baseline was observed as L-arginine concentration increased from 10^−7^M to 10^−5^M and then to 10^−3^M (Fig. 1B). Although our approach of cranial window preparation for superfusion in this study did not permit us to assess the healthy baseline (prior to infection) diameters of arterioles in mice that developed ECM, previous studies^9,23^ applying the classical closed cranial window for monitoring of the brain vasculature during ECM had revealed a vasoconstrictory state of arterioles from healthy baseline levels. This observation coupled with our present findings thus suggested that L-arginine treatment may be capable of reversing or attenuating the vasoconstrictory profile in ECM.

### Pial arteriolar diameter responses to intravenous infusion of L-arginine

It would be of interest to evaluate if systemic introduction of L-arginine via the intravenous pathway is capable of reversing the pial arteriolar vasoconstriction in ECM mice, producing effects similar to those seen with cranial window superfusion.

**Figure 2:**
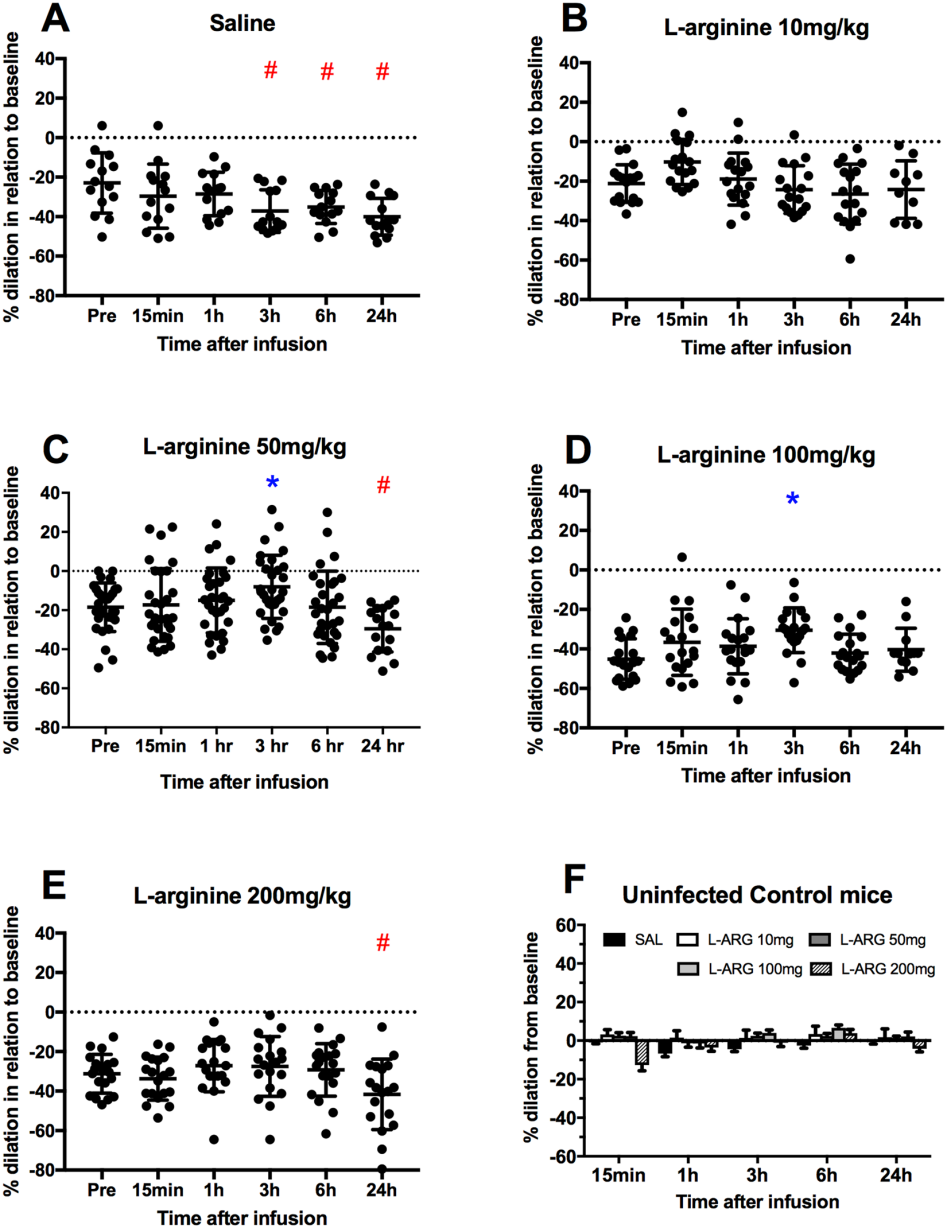
(**A**–**E**) Pial arteriolar diameter responses to L-arginine treatment, given intravenously as a bolus injection, in ECM mice. Each graph represent the responses to different dosages: (**A**) none (saline); (**B**) 10 mg/kg; (**C**) 50 mg/kg; (**D**) 100 mg/kg; (**E**) 200 mg/kg. Four mice were investigated in each dosage group. Each circle represents an individual arteriole (total number per group varied between 14 and 30). Diameter changes are presented relative (%) to their healthy baseline at day 0 prior to infection (dotted line). An asterisk (*) represents a significant increase (p < 0.05) in diameter in relation to time zero (“Pre”), whereas a hashtag (#) represents a significant decrease (p < 0.05) in diameter. (**F**) Pial arteriolar diameter responses to L-arginine treatment, given intravenously as a bolus injection, in uninfected control mice. The bars represent the mean ± standard deviation of % diameter change of each group (saline or L-arginine 10, 50, 100 and 200 mg/kg) relative to pre-treatment.

First, we looked at the pharmacodynamics response of L-arginine in terms of vessel diameter change from pre-treatment levels (relative to day 0 healthy baseline diameters), at various time points (15 min, 1 h, 3 h, 6 h and 24 h) on day 6 of infection. ECM mice treated with saline were used as control. As shown in Fig. 2A–E, substantial arteriolar constriction (between −21% and −35% from day 0 baseline) was generally found in mice with ECM before L-arginine treatment. Arterioles of ECM mice treated with saline not only remained constricted throughout the study period, but actually vasoconstriction worsened at the late time points of 3 h, 6 h and 24 h (going from a mean 22% constriction at pre-treatment to a mean 37% at 3 h and 35% at 6 h). On the other hand, L-arginine treatment, at all dosages, was capable of preventing the aggravation in vasoconstriction at the time points of 3 h and 6 h. Moreover, at the doses of 50 mg/kg and 100 mg/kg, L-arginine was actually capable of ameliorating vasoconstriction, promoting dilation at the 3 h time point (in the case of 50 mg/kg, constriction went from a mean 21% at pre-treatment to a mean 8% at 3 h). At the 24 h time point, constriction worsened in most groups. Responses of individual vessels were variable. For instance, in the case of animals treated with L-arginine at 50 mg/kg, at the 3 h time point, out of 30 arterioles analyzed, 16 dilated, 8 remained relatively stable and 6 showed further constriction in relation to baseline. This response profile was in marked contrast with that observed in ECM mice treated with saline. In this case, at the 3 h time point, out of 14 arterioles analyzed, none dilated, 4 remained relatively stable and 10 showed further and mostly severe constriction. In uninfected mice, L-arginine treatment did not significantly affect vessel diameter at all dosage strengths (Fig. 2F).

**Figure 3:**
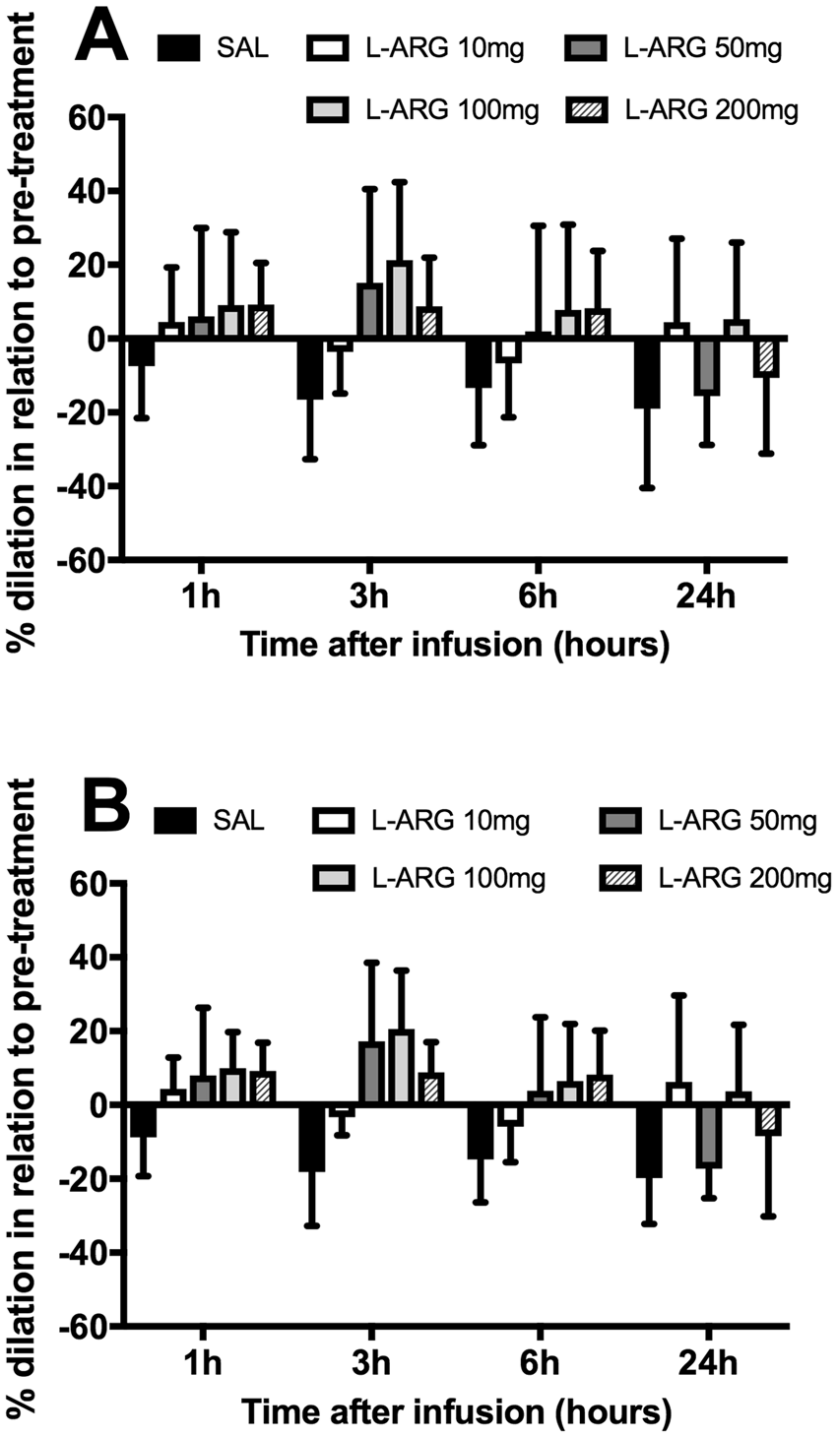
Pial arteriolar diameter responses to L-arginine treatment, given intravenously as a bolus injection, in ECM mice. The bars represent the mean ± standard deviation of % diameter change of each group (saline or L-arginine 10, 50, 100 and 200 mg/kg), in this case relative to pre-treatment (baseline defined as the arteriolar diameters of mice with ECM on day 6, just before treatment). (**A**) Mean ± standard deviation of % diameter change of individual vessels. (**B**) Mean ± standard deviation of % diameter change of individual animals (vessel diameter changes averaged per animal). ECM mice that received saline showed aggravation of vasoconstriction over time (1 h, 3 h, 6 h and 24 h). L-arginine prevented the aggravation of vasoconstriction (at all doses) and even resulted in dilation (doses of 50, 100 and 200 mg/kg). The changes in diameter were significantly different from the saline group for all doses at the timepoints of 1 h, 3 h and 6 h (**A**) (10 mg/kg: p = 0.0097; 50 mg/kg: p = 0.0018; 100 mg/kg: p = 0.0001; 200 mg/kg: p < 0.0001) and for all doses except 10 mg/kg in (**B**) (10 mg/kg: p = 0.1072; 50 mg/kg: p = 0.0435; 100 mg/kg: p = 0.01; 200 mg/kg: p < 0.0186).

As shown, just by comparing vessel diameter changes in relation to their own respective pre-treatment values, the effect of L-arginine treatment seemed rather small. However, when L-arginine treatment at each dose was compared with the saline control in the corresponding time points, a marked effect was evident (Fig. 3). This was true whether the response of individual vessels (Fig. 3A: 10 mg/kg: p = 0.0097; 50 mg/kg: p = 0.0018; 100 mg/kg: p = 0.0001; 200 mg/kg: p < 0.0001) or individual animals (Fig. 3B: 10 mg/kg: not significant, p = 0.1072; 50 mg/kg: p = 0.0435; 100 mg/kg: p = 0.01; 200 mg/kg: p < 0.0186) was accounted for. All four doses proved to be beneficial at the 1 h, 3 h and 6 h timepoints. In terms of magnitude, while an aggravation of vasoconstriction in the order of 16% at 3 hours occurred after saline bolus administration, in ECM mice receiving 50 or 100 mg/kg of L-arginine a vasodilation in the order of 15–21% was observed. There was no significant differences in the responses to the doses of 50, 100 and 200 mg/kg, but each of these three doses induced a stronger response than the 10 mg/kg dose.

### Pial arteriolar diameter responses to continuous subcutaneous delivery of L-arginine

**Figure 4:**
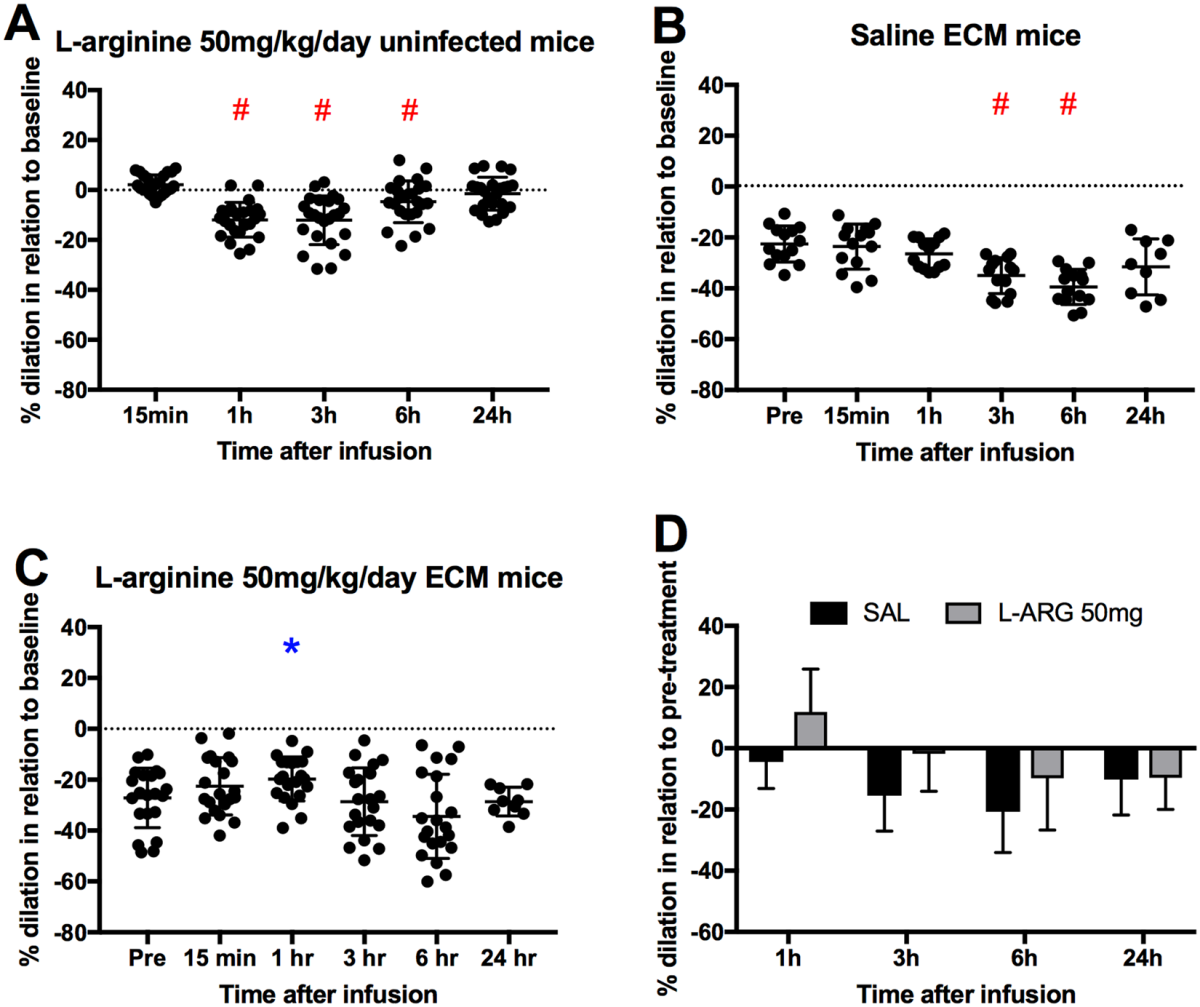
(**A**–**C**) Pial arteriolar diameter responses to slow continuous delivery of L-arginine (50 mg/kg/day) given subcutaneously using osmotic pumps, in uninfected (**A**) and ECM mice receiving saline (**B**) or L-arginine (**C**). Three to four mice were investigated in each group (A: 24 arterioles, B: 14 arterioles, C: 21 arterioles). For uninfected healthy mice, diameter changes are presented with respect to their pre-treatment levels, whereas for infected ECM mice diameter changes are presented with respect to their healthy baseline at day 0 prior to infection. One mouse died in group C after timepoint 6 hours. An asterisk (*) represents a significant increase (p < 0.05) in diameter in relation to time zero, whereas a hashtag (#) represents a significant decrease (p < 0.05) in diameter in relation to time zero. (**D**) Mean ± standard deviation of % diameter change of each group (saline or L-arginine 50 mg/kg/h), in this case relative to pre-treatment (baseline defined as the arteriolar diameters of mice with ECM on day 6, just before treatment). The changes in diameter in the L-arginine group were significantly different from the saline group at the timepoints of 1 h, 3 h and 6 h (p = 0.007).

Small infusion osmotic pumps were implanted subcutaneously on day 6 of infection to provide slow and continuous L-arginine dosing (50 mg/kg/day) of ECM mice over a time period of 24 h. Pial arteriolar diameter changes were monitored at various time points: 15 min, 1 h, 3 h, 6 h and 24 h post treatment. L-arginine treatment of healthy mice served as controls. Interestingly, continuous L-arginine delivery in healthy mice led to vasoconstriction particularly at 3 h and 6 h after treatment. This effect was found to subside with time (Fig. 4A). In mice with ECM, continuous infusion of saline resulted in aggravation of vasoconstriction (Fig. 4B). On the other hand, continuous L-arginine treatment prevented the aggravation in vasoconstriction, and even promoted vasodilation at the 1 h time point, an effect that subsided thereafter (Fig. 4C). Again, compared with saline, continuous L-arginine administration resulted in improved vascular responses (Fig. 4D, p = 0.007).

### Pial arteriolar diameter responses to L-arginine combined with artesunate

**Figure 5:**
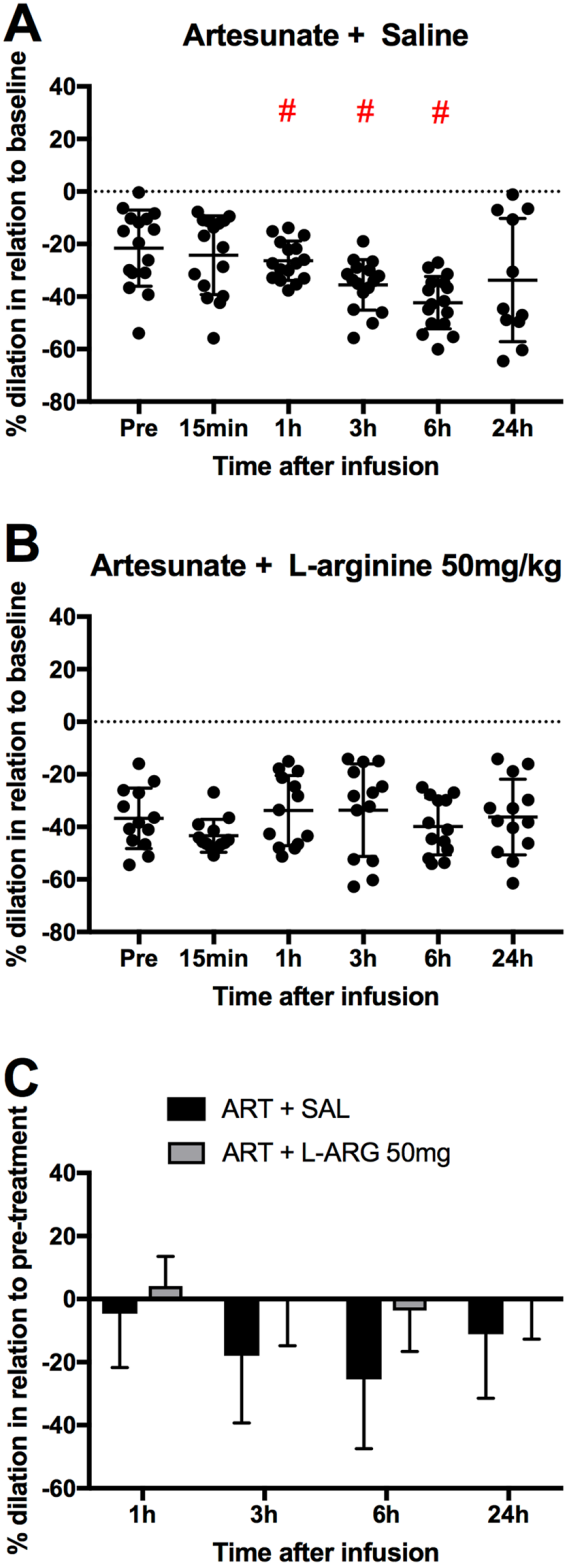
Pial arteriolar diameter responses to combinative therapy of (**A**) saline plus artesunate 32 mg/kg and (**B**) L-arginine 50 mg/kg plus artesunate 32 mg/kg in ECM. Saline and L-arginine were intravenously administered as a bolus injection. Three mice were investigated in each group (A: 16 arterioles, B: 13 arterioles).One mouse died in group A after timepoint 6 hours. An asterisk (*) represents a significant increase (p < 0.05) in diameter in relation to time zero, whereas a hashtag (#) represents a significant decrease (p < 0.05) in diameter in relation to time zero. (**C**) Mean ± standard deviation of % diameter change of each group (saline or L-arginine 50 mg/kg), in this case relative to pre-treatment (baseline defined as the arteriolar diameters of mice with ECM on day 6, just before treatment). The changes in diameter in the L-arginine group were significantly different from the saline group at the timepoints of 1 h, 3 h and 6 h (p = 0.0102).

Our results above revealed that systemic administration of L-arginine at 50, 100 and 200 mg/kg bolus injection intravenously produced the more pronounced effects on reversing the vasoconstriction in ECM mice. Therefore, it would be of interest to evaluate if this beneficial effect on vasomotor tone in ECM mice can be maintained following implementation of an adjunctive therapy approach combining the above L-arginine delivery scheme with artesunate administrated via i.p. ECM mice treated with saline plus artesunate served as control. As shown in Fig. 5, results were similar to those observed when saline or L-arginine were given without artesunate (Fig. 2). Saline together with artesunate failed to demonstrate any reversal in the vasoconstrictory state of ECM mice throughout the observation period (24 h). In fact, pial arterioles became progressively more constricted from pre-treatment (−21.5%) levels, reaching a maximal constriction (−42.3%) at the 6 h time point. In the case of mice with ECM treated with the adjunctive therapy scheme combining L-arginine with artesunate, no aggravation in vasoconstriction was observed (Fig. 5B). A substantial improvement in vascular responses is evidenced when L-arginine versus saline treatments were compared at each timepoint (Fig. 5C, p = 0.0102).

### Survival outcomes of mice treated with L-arginine combined with artesunate

**Figure 6:**
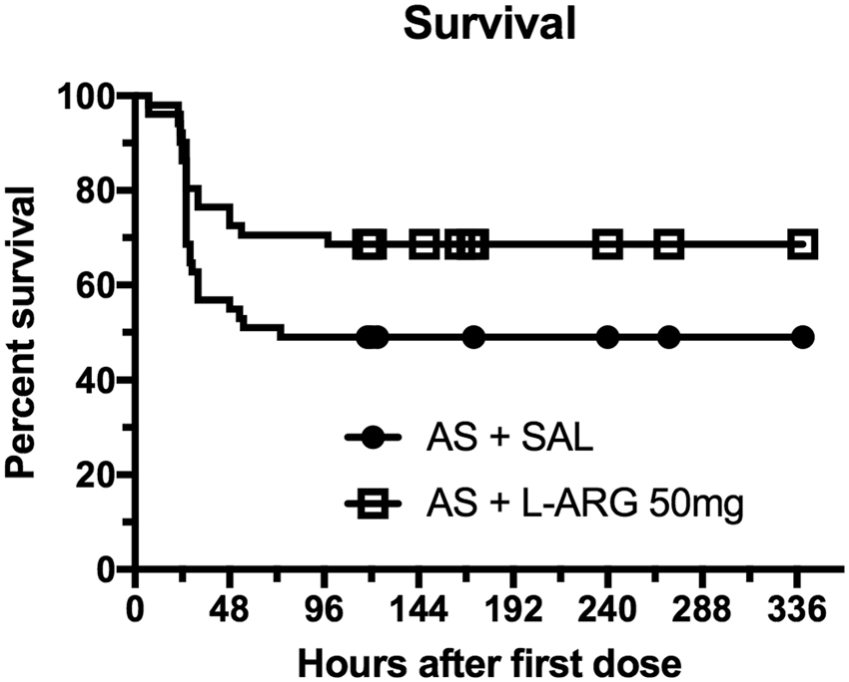
Survival outcomes of mice with ECM treated with artesunate 32 mg/kg (5 doses daily) and either L-arginine 50 mg/kg (AS + L-ARG, n = 51) or saline (AS + SAL, n = 51). Results shown are the combination of four different experiments. Survival was 68.6% in the group that received L-arginine versus 49.0% in the group that received saline (p = 0.047).

Groups of mice presenting ECM were treated with artesunate (32 mg/kg) combined with either L-arginine (50 mg/kg) or saline. Adjuvant therapy with L-arginine resulted in improved survival (68.6% versus 49.0% in the group of mice treated with artesunate plus saline) (Fig. 6, p = 0.047).

## Discussion

L-arginine is a common substrate involved in the regulation of multiple physiological processes. As a precursor of NO production, it is essential for the maintenance of vascular health. In many human pathologies, for instance malaria infection, a state of L-arginine deficiency or hypoarginemia can exist which limits NO production^3^. Underlying causes have been attributed to a plethora of factors such as an increase in enzymatic arginase expression and activation by products of hemolysis and inflammatory reactions^24,25^ as well as inadequate renal L-arginine adsorption or endogenous biosynthesis or recycling^3^. More recently, Alkaitis and coworkers showed that increased arginase activity or impaired protein catabolism do not explain the low plasma L-arginine levels in severe malaria. Instead, decreased L-arginine and citrulline appearance seemed to be primarily responsible for plasma L-arginine and citrulline depletion^5^. A lack of L-arginine abrogates NO production in the brain which in turn can adversely compromise cerebrovascular function. A possible mechanism is the uncoupling of enzymes synthesizing NO, the NO synthases (NOS), which can trigger a vicious cycle of exacerbated oxidative stress (e.g. superoxides and peroxynitrite release) and diminished NO production. We have previously demonstrated that both eNOS and nNOS are dysfunctional in ECM, leading to impaired pial arteriolar reactivity to acetylcholine and N-methyl-D-aspartate (NMDA) stimuli, and shown that the dysfunction can be linked to cofactor BH4 depletion and associated with elevated oxidative stress and NOS uncoupling^22^. These findings, a priori, might suggest a complex picture with multiple factors causing or impacting NOS dysfunction. The remarkable finding of the present study is the demonstration that replenishment of the NOS substrate L-arginine directly to pial vessels causes per se immediate and sustained dose-dependent dilation of constricted arterioles. This finding suggests that a major reason for cerebrovascular dysfunction in ECM can be ascribed to L-arginine deficiency, indicating that otherwise the NOS enzymes are functional even at an environment of oxidative stress and BH4 deficiency, and therefore giving support to the prospect that a relatively simple intervention, that is, L-arginine supplementation, could restore cerebrovascular function in cerebral malaria, as proposed^11^.

This proof of principle is highly relevant, however potential therapies to restore cerebrovascular function in cerebral malaria will not be delivered directly to brain vessels and will have to deal with systemic delivery and all its limitations, including targeting, pharmacokinetics, metabolism, degradation (e.g., oxidation), etc. In human malaria, systemic infusion of L-arginine have shown mixed outcomes. Earlier studies with L-arginine infusion in patients with severe or moderately severe malaria indicated that this intervention was safe and induced improvement in NO bioavailability (measured by exhaled NO levels) and vascular function (measured by reactive hyperemia-peripheral arterial tonometry (RH-PAT)^11,12^. However, in another study with severe malaria patients infusion of L-arginine did not improve lactate clearance or endothelial NO bioavailability^15^. Simulations of dosing schedules using a pharmacokinetic-pharmacodynamic model predicted that increasing the L-arginine dose and using regimens of continuous infusion over longer periods would result in improved efficacy^17^.

In animal models of stroke, intravenous L-arginine infusion promotes nitric oxide-dependent vasodilation and increases regional cerebral blood flow^26,27^. The mouse model provides us with the opportunity to look at the effect of systemic L-arginine supplementation directly on the brain vasculature, which is highly relevant for its expected and presumptive effects in cerebral malaria. The doses chosen (10, 50, 100 and 200 mg/kg) were similar to those used in human studies (the three higher doses equivalent to 3 g, 6 g and 12 g in patients weighing about 60 kg), which showed the existence of a positive correlation between the extent of endothelial function recovery or NO production and ascending doses of L-arginine used for treating patients with moderately severe malaria^11^. In the present study, mice with ECM that received saline, with or without artesunate, showed progressive constriction of pial arterioles over the observation period (24 h), confirming our previous observations with saline-treated or artemether-treated mice^23^. On the other hand, the progression in the intensity of vasoconstriction was halted when L-arginine was given to mice with ECM, with or without artesunate. In addition, at certain doses (especially 50 and 100 mg/kg), L-arginine administration even resulted in transient reversal of vasoconstriction. In terms of magnitude, while an aggravation of vasoconstriction in the order of 16% at 3 hours occurred after saline bolus administration, in ECM mice receiving 50 or 100 mg/kg of L-arginine a vasodilation in the order of 15–21% was observed. This effect, although with some variation in magnitude, was seen whether L-arginine was administered as a bolus, at slow continuous rate or in combination with artesunate. These data provide proof of principle that systemic delivery of L-arginine can partially restore cerebrovascular responses in cerebral malaria. At the dosage used, a continuous subcutaneous delivery system was attempted, but the effect was not better than that observed with the bolus strategy. However, in the case of this continuous, slow delivery system, a dose-dependent response still needs to be established, as reported with nimodipine and glyceryl trinitrate^23,28^. In severe malaria patients, no significant effect of L-arginine treatment given as slow infusion (1.5 g/hr over 8 hrs) on lactate clearance and NO production was observed^15^. The lack of pharmacological response of L-arginine treatment was attributed to the low concentration of L-arginine administered coupled with greater severity of the disease condition, such that the biologically available quantity of L-arginine may not be pharmacologically effective which otherwise highlighted the need for a higher dosage regime to demonstrate efficacy. Also, in the human studies of L-arginine infusion in malaria patients, there was some concern of decreases in bicarbonate and pH due to the hydrogen and chloride ion components of the L-arginine hydrochloride, the only formulation available for human use^15,29^. The present study was performed with a pure, non-hydrochloride-containing, L-arginine, which may have resulted in improved responses.

Data from moderately severe malaria patients indicates that increasing the L-arginine dose coupled with infusion regimens over longer periods may improve the vascular effects of L-arginine^17^. In animal studies, a much higher dose (1,500 mg/kg) resulted in exacerbation of cerebral malaria, with earlier mortality^19^. However, in that case, L-arginine was given as a preventative treatment, before or just after starting the infection, and therefore it is not comparable to this study design. In similar preventative treatment studies with lower doses of L-arginine, the exacerbation of the disease was not observed^18^.

In the present study, although the outcome in terms of vascular diameters showed marked improvement in L-arginine-treated as compared to saline-treated ECM mice, L-arginine either as monotherapy or combined with artesunate showed variable responses, reversing constriction or inducing dilation in most but not all vessels or animals. Further studies are necessary to understand the factors involved in these variable responses and optimize the benefit of L-arginine supplementation on cerebrovascular health.

When comparing saline-treated versus L-arginine-treated ECM mice, the beneficial effects on vascular diameters peaked at 3 hours and persisted at 6 hours. Alternative treatment protocols might be taken into consideration, for instance with the inclusion of additional doses to ensure even more prolonged and sustained responses. However, even a temporary amelioration of cerebrovascular responses might possess tremendous impact in patient recovery, as it would help to maintain brain function and life at the most critical period, the time window between the patient’s admission and before the antimalarial drug effectively kill the parasites, buying the patient a precious few hours. This interpretation is strengthened by the finding that L-arginine supplementation in combination with artesunate resulted in improved survival in relation to mice treated with artesunate combined with saline. Therefore, despite the limitations of systemic delivery, L-arginine not only helped restoring cerebrovascular function but had a benefit on survival, which is the critical outcome for any adjunctive therapy.

L-arginine supplementation has been tested or considered in a number of pathological conditions such as angina pectoris, congestive heart failure, erectile dysfunction, sickle cell disease and others. Its potential for treating chronic conditions, however, has been challenged by reports of “L-arginine tolerance”, as its benefits on vascular function were not seen after long term exposure^30,31^. *In vitro* studies showed that longer term exposure to L-arginine was actually detrimental instead of beneficial to vascular health, by suppressing endothelial NOS activity and intensifying oxidative stress levels^32^. However, in acute situations such as cerebral malaria, L-arginine will be administered during a short time window lasting hours or a few days, between admission/start of treatment and recovery from coma, and therefore this type of tolerance is not expected to occur.

Overall, the present study provides evidence and proof of principle that L-arginine supplementation is capable of partially reversing cerebrovascular constriction in experimental cerebral malaria, or at least prevent its worsening after antimalarial treatment is initiated. However, while topical superfusion produces consistent dilatory responses in pial vessels, systemic intravenous infusion results in variable and dose-dependent responses. Further studies are necessary to optimize the systemic delivery schemes that result in more consistent and sustained vascular responses. The definition of such optimized schemes could provide critical information for such optimization also in human severe malaria L-arginine-based therapies.

## Materials and Methods

### Infection of mice

All protocols were carried out in accordance with relevant guidelines and regulations and were approved by the La Jolla Bioengineering and Fiocruz Institutional Animal Care and Use committees. Eight-to-ten-week-old female C57BL/6 mice (Jackson Laboratories, Bar Harbor, ME) were intraperitoneally (i.p.) inoculated with 10^6^ *Plasmodium berghei* ANKA (PbA) parasites expressing the green fluorescent protein (PbA-GFP, a donation from the Malaria Research and Reference Reagent Resource Center - MR4, Manassas, VA; deposited by CJ Janse and AP Waters; MR4 number: MRA-865).

### Clinical parameters

To measure the parasitemia, a small blood sample (~1 µl) was obtained by a mouse tail end prick, and flow cytometry was used to detect and count the number of parasitized RBCs that expresses GFP in relation to 10,000 RBCs. For rectal temperature measurement, a thermocouple probe (Oakton® Acorn^TM^; Oakton Instruments, IL, USA) was used. Motor behavioral score was determined by a composite scoring system based on six motor behavior tests modified from the SHIRPA protocol^33^. ECM was defined as the presentation of one or more of the following clinical signs of neurological involvement: ataxia, limb paralysis, poor righting reflex, seizures, roll-over and coma.

### Window preparation for cranial superfusion

A cranial window preparation scheme with improved stability to deliver test compounds to the brain of ECM mice was adopted here^21^. Briefly, highly invasive surgical procedures (skin removal and skull drilling) typically involved in a craniotomy were performed beforehand in the healthy animal to create a skull bone flap prior to infection of the animal to induce ECM. At the time of experimentation, the bone flap can be readily retracted and a prefabricated perfusion chamber can be assembled to enable superfusion of its exposed brain cortical surface. This approach minimizes mechanical trauma exerted on the delicate brain tissue of the sick animal during window preparation for study. The same window preparation procedures were applied to the healthy uninfected mice that serve as control in all superfusion experiments.

### Closed window preparation for chronic imaging of vessel diameter

The classical closed cranial window preparation, as previously described^34,35^, was utilized for monitoring long term changes in pial arteriolar diameters. This type of window preparation, unlike the window for cranial superfusion, does not allow direct topical delivery of test compounds to the brain cortex and therefore, test agents will have to be delivered systemically. In the present study, test agents were delivered via the intravenous or subcutaneous pathway. On the other hand, this type of window preparation allows repeated measurements to be performed at the same site in the cerebrovasculature, and therefore, it is advantageous for prolonged monitoring of the same vessel throughout the course of the disease genesis. By utilizing this approach, we are able to monitor changes in vessel diameter of the same arteriole from pre-infection to ECM development and then from pre-treatment to post-treatment with L-arginine delivered systemically.

### Intravital microscopy and vessel diameter quantification

Mice implanted with cranial window (superfusion/closed type), were mildly anesthetized with isofluorane and transferred onto an intravital microscope stage (customized Leica-McBain, San Diego, CA). Randomly chosen pial arterioles (N = 2–6 per mouse; baseline vessel diameters = 35 to 115 µm) distinguished by their diverging flow pattern at bifurcating points throughout the vasculature, were visualized by epi-illumination using a 20_water immersion objective lens, and their diameters measured using an Image Shear device (0.213 _m/pixel; Vista Electronics, San Diego, CA).

### L-arginine superfusion procedures

Both uninfected healthy and infected ECM mice, implanted with cranial windows for superfusion, were subjected to the L-arginine superfusion protocol. Accordingly, pial arterioles were visualized through the cranial window using intravital microscopy, and changes in diameter were recorded after superfusion with L-arginine. L-arginine was introduced in sequence of increasing concentration (10^−7^M to 10^–3^M) with a superfusion duration of 30 mins for each concentration which ensured that steady state vessel diameter was achieved before the next concentration was delivered.

### L-arginine systemic treatment procedures

Two weeks after surgery, baseline diameter of pial arterioles in uninfected mice with implanted closed cranial windows were acquired. Spatial coordinates or visual landmarks allowing repeated easy tracking of the same vessel location were noted. Mice were then infected i.p. with PbA (10^6^), and those that developed ECM (specifically, rectal temperatures between 32–36 °C) on day 6 of infection were subjected to intravital microscopy to obtain diameters of the same arterioles. Following which, mice were subjected to the treatment protocol and vessel diameters were again acquired at multiple time points (0, 3, 6, and 24 hr) after treatment. Treatments were introduced systemically in two ways, either as a bolus injection through the mouse tail vein or through continuous delivery via the subcutaneous pathway using Alzet osmotic pumps (see description below). For each administration pathway, mice were treated with L-arginine (Tocris Bioscience, Ellisvile, MO, USA) or saline (as control). L-arginine was also combined with artesunate (Sigma, St Louis, MO, USA, 32 mg/kg/day) injected i.p. to assess the effect of this combined therapy on pial arteriolar diameter response. L-arginine was dissolved in saline whereas artesunate was dissolved in 5% sodium bicarbonate in saline. Another group of mice, correspondingly treated with saline plus artesunate, served as control. Study for each treatment group lasted for 24 hours, after which the mice were euthanized with a cocktail of sodium pentobarbital at 390 mg plus sodium phenytoin at 50 mg/ml (Euthasol; 100 mg/kg, i.p.).

### Continuous L-arginine delivery via osmotic pumps

To achieve continuous delivery of pharmacological agent systemically, osmotic pumps (Alzet, Cupertino, CA) were implanted subcutaneously in healthy/ECM mice. Osmotic pumps (model 1003D, constant delivery rate of 1 µL/hour for up to 3 days) were filled with the appropriate solution (42 mg/mL of L-arginine to target a delivery of 50 mg/kg/day of L-arginine or saline as control – 100 µL final volume) and primed in 0.9% sterile saline at 37 °C for approximately 4 hours to ensure immediate delivery of the contents after implantation. Mice with ECM (day 6 of infection) were anesthetized using isofluorane and the primed pumps were implanted subcutaneously in the back, slightly posterior to the scapulae, under sterile conditions.

### Survival experiments

These experiments were designed to determine whether L-arginine had a beneficial effect when given as adjunctive therapy in combination with artesunate in mice with ECM, as previously described^9,23^. Development of cerebral malaria was assessed by clinical evaluation of neurological signs such as ataxia, convulsions, limb paralysis and/or coma. Rectal temperature was used as the objective criterion for treatment, as mice with cerebral malaria develop hypothermia. Mice presenting rectal temperature in the range of 32–36 °C were randomly assigned to two groups: 1) treated with artesunate 32 mg/kg in 5% bicarbonate solution (intraperitoneal) plus L-arginine 50 mg/kg in saline (subcutaneously); 2) treated with artesunate 32 mg/kg in 5% bicarbonate solution (intraperitoneal) plus L-saline (subcutaneously). All mice received artesunate once daily for 5 days. L-arginine or saline were given in two doses, at time zero and 24 hours after first dose. Mice were followed up for 7 days after the last dose and then euthanized with overdose of pentobarbital.

### Statistical analyses

All statistical analyses were performed using a statistical software package (Prism 7, Graphpad). To compare three or more experimental groups in terms of temperature, motor score and parasitemia, a one-way ANOVA test with Bonferroni pos hoc analysis was applied. To determine the effect of treatment on arteriolar diameter response in ECM mice, arteriolar diameter change relative to day 0 healthy baseline was compared between pre-treatment and post-treatment (intra-group) at individual time points (15 mins, 1 hr, 3 hr, 6 hr or 24 hr) using two-tailed paired t-test. A two-way ANOVA was performed to determine the effect of treatment (each L-arginine dose) and time (1 hr, 3 hr and 6 hr–24 hr was not analyzed as some of the mice died) in relation to the control saline group. Analyses were performed considering either the individual vessels or the individual animals (in which case the responses of individual vessels of each animal were averaged). All reported data were in mean ± SD. Survival curves were analyzed with Log-rank test. P < 0.05 was considered statistically significant.
